# Neutrophil depletion in the pre-implantation phase impairs pregnancy index, placenta and fetus development

**DOI:** 10.3389/fimmu.2022.969336

**Published:** 2022-09-29

**Authors:** Cristina Bichels Hebeda, Anna Carolina Savioli, Pablo Scharf, Marina de Paula-Silva, Cristiane Damas Gil, Sandra Helena Poliselli Farsky, Silvana Sandri

**Affiliations:** ^1^ Department of Clinical and Toxicological Analyses, School of Pharmaceutical Sciences, University of São Paulo, SP, São Paulo, Brazil; ^2^ Núcleo de Pesquisa em Ciências Médicas, Fundação Universidade para o Desenvolvimento do Alto Vale do Itajaí – UNIDAVI, Rio do Sul, SC, Brazil; ^3^ Center for Stem Cells and Regenerative Medicine, King’s College London, London, United Kingdom; ^4^ Department of Morphology and Genetics, Federal University of São Paulo, São Paulo, SP, Brazil

**Keywords:** anti-granulocytes, pregnancy index, abnormal fetal development, maternal immune system, placenta morphology, window of implantation

## Abstract

Maternal neutrophils cells are players in gestational tolerance and fetus delivery. Nonetheless, their actions in each phase of the pregnancy are unknown. We here investigated the role of maternal neutrophil depletion before the blastocyst implantation phase and outcomes in the pregnancy index, placenta, and fetus development. Neutrophils were pharmacologically depleted by i.p. injection of anti-Gr1 (anti-neutrophils; 200 µg) 24 hours after plug visualization in allogeneic-mated C57BL/6/BALB/c mice. Depletion of peripheral neutrophils lasted until 48 hours after anti-Gr1 injection (gestational day 1.5-3.5). On gestational day 5.5, neutrophil depletion impaired the blastocyst implantation, as 50% of pregnant mice presented reduced implantation sites. On gestational day 18.5, neutrophil depletion reduced the pregnancy rate and index, altered the placenta disposition in the uterine horns, and modified the structure of the placenta, detected by reduced junctional zone, associated with decreased numbers of giant trophoblast cells, spongiotrophoblast. Reduced number of placenta cells labeled for vascular endothelial growth factor (VEGF), platelet-endothelial cell adhesion molecule (PECAM-1), and intercellular cell adhesion molecule (ICAM-1), important markers of angiogenesis and adhesiveness, were detected in neutrophil depleted mice. Furthermore, neutrophil depletion promoted a higher frequency of monocytes, *natural killers*, and T regulatory cells, and lower frequency of cytotoxic T cells in the blood, and abnormal development of offspring. Associated data obtained herein highlight the pivotal role of neutrophils actions in the early stages of pregnancy, and address further investigations on the imbricating signaling evoked by neutrophils in the trophoblastic interaction with uterine epithelium.

## Introduction

Mammalian reproduction is a complex phenomenon, which generally leads to inefficient outcomes. The majority of conceptions do not evolve, especially by losses before the blastocyst implantation (1, 2), which takes place in a very short period of gestation named window of implantation (WOI). During WOI occurs the synchronization of embryonic development and uterine differentiation into a receptive status (3). WOI is characterized by pre-receptive and receptive periods followed by refractory no receptive phases. In humans and rodents, implantation of blastocysts begins with apposition, followed by attachment to trophoblast outgrowth and decidualization, which are spatiotemporally regulated by many players, such as endocrine/growth factors/immune mediators of cell-cell and cell-matrix interactions (3, 4). Disruptions to the implantation process are also considered causes of pregnancy-associated complications, such as preeclampsia, intrauterine growth restriction, and preterm birth (5, 6).

The maternal immune response in the endometrium is pivotal to the establishment of WOI. The fetal antigen contact with the maternal endometrium modifies the local repertoire of immune cells, which display a fundamental role in trophoblast invasion, vascular remodeling by extravillous trophoblast, embryo invasion, and maternal tolerance (7, 8). The innate immune response in the blastocyst implantation period is not characterized as classical inflammation, as it occurs to enhance the adhesiveness of the uterine epithelium and the formation of new vessels for the blood supply. Therefore, innate-mediated inflammation must be local and adjustable to the process, as both impaired and exacerbated innate response leads to failure in the blastocyst-endometrium interactions (9).

Neutrophils are the predominant circulating innate immune cells in humans prompted to migrate into the site of inflammation to eliminate insulting agents. More recently, the role of neutrophils in host defense and homeostasis has been outspread by identifying neutrophil phenotypes, which display pro or anti-inflammatory properties and influence the resolution of inflammation and tissue repair (10). Neutrophil switching to distinct subtypes is dependent on the phase of the inflammatory process and modulated by the chemical and cellular composition of the surrounding inflammatory microenvironment (11).

In this context, it has been highlighted that neutrophils acquire different phenotypes during pregnancy, with fundamental roles in each phase. Studies describing neutrophils phenotype in the placental microenvironment are scarce. However, all studies corroborate that neutrophils display a proangiogenic phenotype. In the first trimester, neutrophils are found in the human decidua, in an area mainly involved in tissue remodeling and physiological decidual implant reaction, displaying high resistance to apoptosis, do not exerting a suppressive activity, and expressing fibro/angiogenic factors (12) In the second trimester, the number of neutrophils at decidua basalis is increased and high levels of activation markers and angiogenesis-related proteins, such as vascular endothelial growth factor-A (VEGF) are detected (13). In pregnant mice, the augmented neutrophils numbers were observed on gestational days 8 – 10, which coincides with the period of spiral arterial angiogenesis (13).

Several chemical mediators in the uterine microenvironment modulate neutrophil recruitment and functional phenotypes in normal pregnancy (14–16). Studies have demonstrated that placental cell debris and IL-8 are involved in the neutrophil migration to decidua (17). During coitus, inflammatory neutrophils are recruited for the elimination of excessive sperm through phagocytosis; nevertheless, in the following phases, they are inhibited to allow the healthy motile sperm to reach the oviduct (18). Moreover, neutrophils are found in the uterine stroma at the early phase of WOI and proangiogenic neutrophils were identified in the second‐trimester decidua of normal pregnancy, displaying the ability to perform transendothelial invasion and induce endothelial tube formation *in vitro* in comparison to resting circulating neutrophil (13, 15). During maternal tolerance mediated by T cells, mice neutrophils in the pregnant uterus acquire an anti-inflammatory phenotype, undergo apoptosis, and release intracellular granules into T cells, favoring the Treg maternal tolerance, and placenta angiogenesis; the frequency of anti-inflammatory neutrophils is reduced in the blood of pre-eclampsia mothers (19). During childbirth, local neutrophils are classical pro-inflammatory cells, which contribute to fetus expulsion (20). Moreover, it is evident that pro-inflammatory activated neutrophils are involved in the pre-eclampsia pathogenesis, and a higher neutrophil-lymphocyte ratio is related to preterm labor (7, 18, 19, 21).

Some studies using antibody-mediated circulating neutrophil depletion approaches have highlighted the role of these cells in the placenta and fetus development. In this context, Higashisaka and collaborators showed that neutrophil depletion in mice on gestational day 15 just exacerbated the pregnancy complications induced by the silica nanoparticles, being that neutrophil depletion per se was not able to impair the pregnancy outcomes (22). On the other hand, Nadkarni and collaborators showed that neutrophil depletion on gestational days 5 and 8 promoted abnormal placentation and fetus alterations (19). These studies suggest that the effects of neutrophil depletion can be associated with the earlier stages of placentation. Indeed, we show that circulating neutrophils are required for efficient placentation and fetal development.

## Material and methods

### Animals and allogenic pregnancy

Mice were maintained and reproduced at Animal House at the School of Pharmaceutical Sciences, University of Sao Paulo (Brazil) with chow and water *ad libitum* in a temperature-controlled room (22–25°C and 70% humidity) with a 12-hr light-dark cycle. Allogeneic pregnancy was performed by mating male Balb/C with female C57Bl/6 mice (both 5–6 weeks old). Females were caged overnight with males (3:1), and successful mating was verified the following morning by the presence of a vaginal plug. All procedures were performed according to the Brazilian Society for the Science of Laboratory Animals (SBCAL) and approved by the Institutional Animal Care and Use Committee from the Faculty of Pharmaceutical Sciences of the University of Sao Paulo (Protocol number 557).

### Peripheral maternal neutrophil depletion

Neutrophil depletion was achieved by intraperitoneal injection of 200 µg anti-Gr1 antibody (clone RB6-8C5; e-Bioscience/Invitrogen, Waltham, Massachusetts, USA), isotype-matched control antibody (clone eB149/10H5; Rt IgG2b k; e-Bioscience/Invitrogen), or phosphate-buffered saline (Control) into C57Bl/6 pregnant at 24 h after vaginal plug visualization (reported as gestational day 0.5). Maternal blood was collected from the abdominal aorta and used to perform leukogram and flow cytometry.

### Peripheral leukocyte profile and immunophenotyping by flow cytometry

The effectiveness of maternal peripheral neutrophil depletion and peripheral leukocyte profile was evaluated by total and differential leukogram, and flow cytometry. For the evaluation of maternal peripheral neutrophil depletion, peripheral blood was collected by aortic puncture in tubes containing heparin on gestational day 3.5. The leukogram was performed under optical microscopy by manual counting in the Neubauer chamber using Turk’s solution. The morphological evaluation was performed using Panotico staining smears (peripheral blood mononuclear cells - MNs and peripheral polymorphonuclear cells - PMNs).

Flow cytometry was employed to confirm the efficacy of anti-Gr1 injection using peripheral blood collected by aortic puncture in tubes containing heparin. Leukocyte population, PMNs and MNs were isolated using forward versus side scatter channels.

Peripheral leukocyte profile was investigated by flow cytometry. Leukocytes were obtained from abdominal aorta on gestational day 18.5. Whole blood was lysed with an ammonium lysis buffer. Isolated leukocytes were incubated with PE-anti-mouse CD3; FITC anti-mouse CD4, APC anti-mouse CD8, APC anti-mouse FoxP3, PE anti-mouse B220; FITC anti-mouse F4/80; PerCP Cy5 anti-mouse-Gr1, and FITC anti-NK1.1 (BD-Bioscience or Invitrogen) for 50 minutes at room temperature and in the dark. In sequence, the labeling cells were acquired in an Accuri C6 flow cytometer (BD Biosciences), and 10,000 events were considered for analysis. The data were expressed as the frequency of positive cells.

### Cesarean section procedure

C-sections were performed under aseptic conditions on gestational days 5.5 to count the number of implantation sites and on 18.5 to investigate the reproductive and pregnancy outcomes and to collect the fetus and placentas for posterior analysis. C-sections were performed after mice were anesthetized with xylazine and ketamine (7 mg/kg and 77 mg/kg, respectively; i.p; Vetbrands, Jacarei, SP, Brazil). In addition, on gestational day 18.5, maternal blood was collected from the abdominal aorta and used to identify the leukocyte profile by flow cytometry.

### Reproductive and pregnancy outcomes calculation

On gestational day 5.5, the uterus was surgically removed for implantation points count. The pregnancy index and outcomes were performed as previously described (23, 24). Briefly, the successful pregnancy index was calculated considering the ratio of mice that presented vaginal plugs and the presence of live fetuses at C-section performed on gestational day 18.5. Also, the uterus and ovary were removed followed by the count of fetuses and resorption points. The uterus, placenta, and fetus were also weighted, and a placenta index was calculated by considering the ratio between the weight of the fetus and the weight of their respective placentas.

### Histological procedures and analyses

Placentas were fixed in 4% buffered paraformaldehyde and histologically processed for inclusion in paraffin. Sections of 4 μm were stained according to analyses performed. The photomicrographs were obtained by optical microscopy using high-power objectives on the Imager.A2 Zeiss microscope (Zeiss, Oberkochen, Germany). The quantification of all the parameters cited below was carried out using ImageJ software by analyzing 5 fields of each section.

### Placenta morphometric analysis

Placenta morphometry (total size, decidua, junctional zone, and labyrinth area) was measured in hematoxylin and eosin-stained (H&E) sections using the Axiovision software (Zeiss, Oberkochen, Germany). Periodic Acid Schiff (PAS) staining was performed using a commercial kit and following the manufacturer’s instructions (EasyPath; São Paulo, Brazil) to count the glycogen-positive cells. Also, the giant trophoblast cells and spongiotrophoblast were counted based on morphologic characteristics. The cell count was performed using the Cell counter tool from Image J software (National Institutes of Health, Bethesda, USA). Five fields were analyzed in the junctional zone from each placenta section by two independent observers.

### Immunohistochemistry

After placenta section permeabilization (0.01% Triton X; Sigma) (Sigma-Aldrich, Burlington, USA) and antigen retrieval (sodium citrate buffer, 10 mM, pH 6.0, 30 minutes in 96°C), endogenous peroxidase was blocked with three rounds of 3% hydrogen peroxide. Slides were incubated with blocking buffer (5% Tris-buffered saline–bovine serum albumin; 1 h) and incubated overnight with purified rabbit anti-VEGF (1:200; Invitrogen) goat anti-ICAM, or anti-PECAM antibodies (both 1:25; Santa Cruz Biotechnology, Dallas, USA). Thus, the slides were washed with tris-buffered saline three times and then incubated with anti-rabbit (1:250; Abcam, Cambridge, UK) or anti-goat (1:100; R&D System, Minneapolis, USA) horseradish peroxidase (HRP) antibody, followed by 3,3-diaminobenzidine (DAB; DAKO, Glostrup, DEN), and hematoxylin counterstaining. As negative control of reactions, slides were incubated only with anti-rabbit or anti-goat HRP antibodies. 

### Statistical analysis

Data were analyzed using Student’s t-test or one-factor analysis of variance (One-Way ANOVA) followed by Tukey *post-hoc* test using GraphPad software version 7. Differences between assessed means were considered statistically significant assuming p < 0.05. All results are shown as mean ± standard error of the mean. When needed, nominal data were statistically evaluated using the Fischer Exact Test and p < 0.05 was considered statistically significant.

## Results

### Neutrophils depletion before the implantation phase reduced the pregnancy index

**Figure 1:**
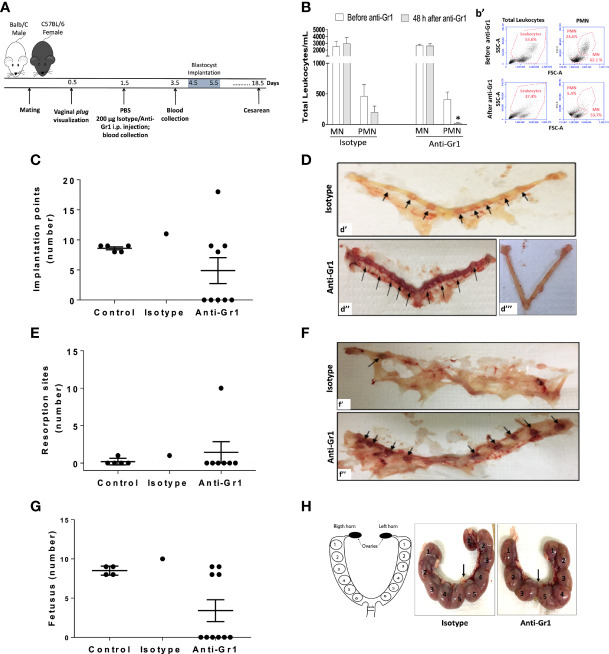
Neutrophil depletion before the implantation phase of pregnancy reduces pregnancy index and reproductive outcomes. Anti-Gr1/isotype injection and the subsequent endpoints were illustrated in the experimental workflow **(A)**. The total number of mononuclear cells (MN) and polymorphonuclear (PMN) was monitored before and after 48h of anti-Gr1/isotype i.p. injection by manual count **(B)** and flow cytometry **(b’)**, respectively. On gestational day 5.5, mice were euthanized for implantation points count **(C)**. Representative images of implantation points **(D** - arrows**)** in the isotype **(d’)**, and in the Anti-Gr1 **(d’’)** groups are shown. The lack of implantation points observed in anti-Gr1 treated mice is also depicted **(d’’’)**. On gestational day 18.5, pregnant mice were submitted for cesarean and the number of resorption points **(E)**, fetuses **(G)**, and disposition of fetuses in the uterine horns **(H)** was analyzed. Representative images of resorption sites **(F)** in the isotype **(f’)** and Anti-Gr1 **(f’’)** are shown. Schematic diagram, isotype, and Anti-Gr1 placenta distribution into the uterine horns are demonstrated **(H)**. Each number indicates a fetus inside of the placenta. Arrow indicates cervix location. Data were statistically analyzed using One-Way ANOVA followed by Tukey’s post-test (n= 5 – 9 animals/group) and p < 0.05 was considered statistically significant. * p < 0.05 versus respective control before Anti-Gr1 injection **(B)**. No significant difference was observed **(C, E, G)**.

Neutrophil depletion at the pre-receptive phase of implantation, was carried out by i.p. of anti-Gr1 24 hours after vaginal plug visualization (**Figure 1A**). In this experimental approach, a decrease in the circulating neutrophils number was observed up to 48 hours after anti-Gr1 i.p injection, while the mononuclear cells population was not affected (**Figures 1B** and **b’**). The depletion of circulating neutrophils abrogated implantation points in 50% of the pregnant mice, as observed by laparotomy performed on gestational day 5.5. Differently, all control animals (100%) presented around ten points of implantation. It is important to mention that the remaining 50% of mice treated with anti-Gr1, presented around eight and just one had almost twenty points of implantation (**Figures 1C, D**). On gestational day 18.5, pregnant mice were submitted for cesarean section and reproductive parameters were analyzed. Herein, it was observed that the number of resorption sites in neutrophil-depleted mice was similar to those found in the control/isotype groups (**Figure 1E**) Ilustrative image of the absence of resorption sites is exhibited in the **Figure 1F - f'**. Only one anti-Gr1 animal presented higher resorption sites as shown in **Figure 1 - f''**. Nonetheless, the pregnancy failure rate was around 60% in the neutrophil-depleted group. On the other hand, the control/isotype group showed a rate of pregnancy success of around 86% (**Supplementary Table 1**). Furthermore, in the neutrophil-depleted mice that had a successful pregnancy, the number of the fetuses was similar to the control group (**Figure 1G**), and the weight gain of pregnant mice and the weight of the pregnancy uterus were not altered (**Supplementary Figures 1A, B**). The gross analysis of the uterus showed that neutrophil depletion altered the placenta’s disposition inside of uterine horns. As shown in **Figure 1H**, the control/isotype mice showed the same number of placenta on both uterine horns (four fetuses on each horn - left/right). On the other hand, neutrophil-depleted mice presented three and five placentas on the right and left horns, respectively, showing no equidistant pattern as observed for the control/isotype group. Moreover, the placental weight and index were not changed (**Supplementary Figures 1C, D**).

### Neutrophils depletion impaired the development of the junctional zone

**Figure 2:**
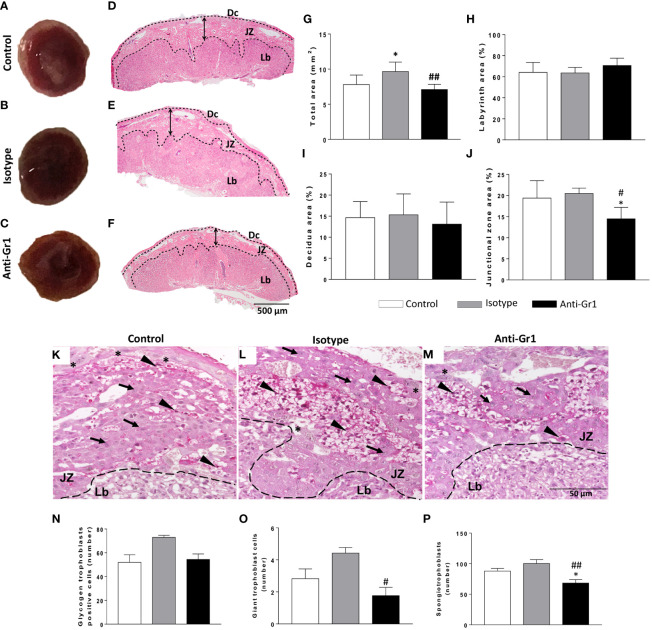
Neutrophil depletion altered placenta morphology and reduced the number of specialized cells in the junctional zone. On gestational day 18.5, placentas were removed for gross analyses **(A-C)** and then submitted for histology evaluation with H&E staining **(D-F)**. Morphometrical analysis was employed to measure the total area **(G)**, the Lb- labyrinth **(H)**, Dc - decidua **(I)**, and JZ - junctional zone **(J)** using PAS staining. The count of the types of cells in the junctional zone and the presence of glycogen trophoblast-positive cells was performed by analyzing five fields **(M)**. The trophoblast subtypes were quantified and indicated in the sections as glycogen-trophoblast cells **(N)**, giant trophoblast cells **(O)**, and spongiotrophoblast **(P)**. * < 0.05 in comparison to control group; #p < 0.05; ##p < 0.01 in comparison to isotype control. Data were statistically analyzed using One-Way ANOVA followed by Tukey’s post-test (n = 6 – 9). Results were expressed as mean ± SEM. Arrowheads in PAS staining **(K-M)** indicate spongiotrophoblast (SpTs); arrows indicate glycogen trophoblasts (GlyTCs), and asterisks indicate giant trophoblast cells (TGCs). Embedding paraffin; sections thickness 4 µm. Bars 50 µm.

Macroscopic analysis of the placenta from neutrophil-depleted mice showed a reduced size and altered macrostructure compared to the control/isotype group (**Figures 2A–C**). Thus, morphometric analyses were performed. Placenta sections stained with H&E were used to measure the total area and the three main placental layers: the labyrinth (Lb), the junctional zone (JZ), and the maternal decidua (Dc) (**Figures 2D–F**). As shown in **Figure 2G**, the neutrophil-depleted mice showed a reduced total area in comparison to control/isotype groups. The labyrinth and decidua areas were not altered by neutrophil depletion (**Figures 2H, I**). However, it was verified that anti-Gr1 treatment decreased the junctional zone area (**Figure 2J**). Considering the role of the junctional zone as an endocrine compartment and potential energy source (25, 26), we evaluated whether the number of cell types that comprise this layer (glycogen trophoblast cells - GlyTCs; spongiotrophoblast - SpT; and trophoblast giant cells - TGCs) was altered. As shown in **Figures 2K–M**, at the junctional zone, it was verified the presence of GlyTC (arrow) exhibiting vacuoles containing particles of glycogen (PAS positive); TGCs (*) characterized by the largest cytoplasm and located at the boundary between JZ and Dc zones; and SpTs (arrowhead) identified as clusters of tightly packed polygonal cells that comprise the middle layer of the placenta sandwiched between the outer secondary TGCs and the Lb layer. Further, we observed that the number of GlyTC was not significantly altered by neutrophil depletion (**Figure 2N**). However, we verified that the counting of TGCs and SpT was decreased in neutrophil-depleted mice (**Figures 2O, P**).

### The angiogenesis and adhesiveness markers in the junctional zone were altered by neutrophils depletion

**Figure 3:**
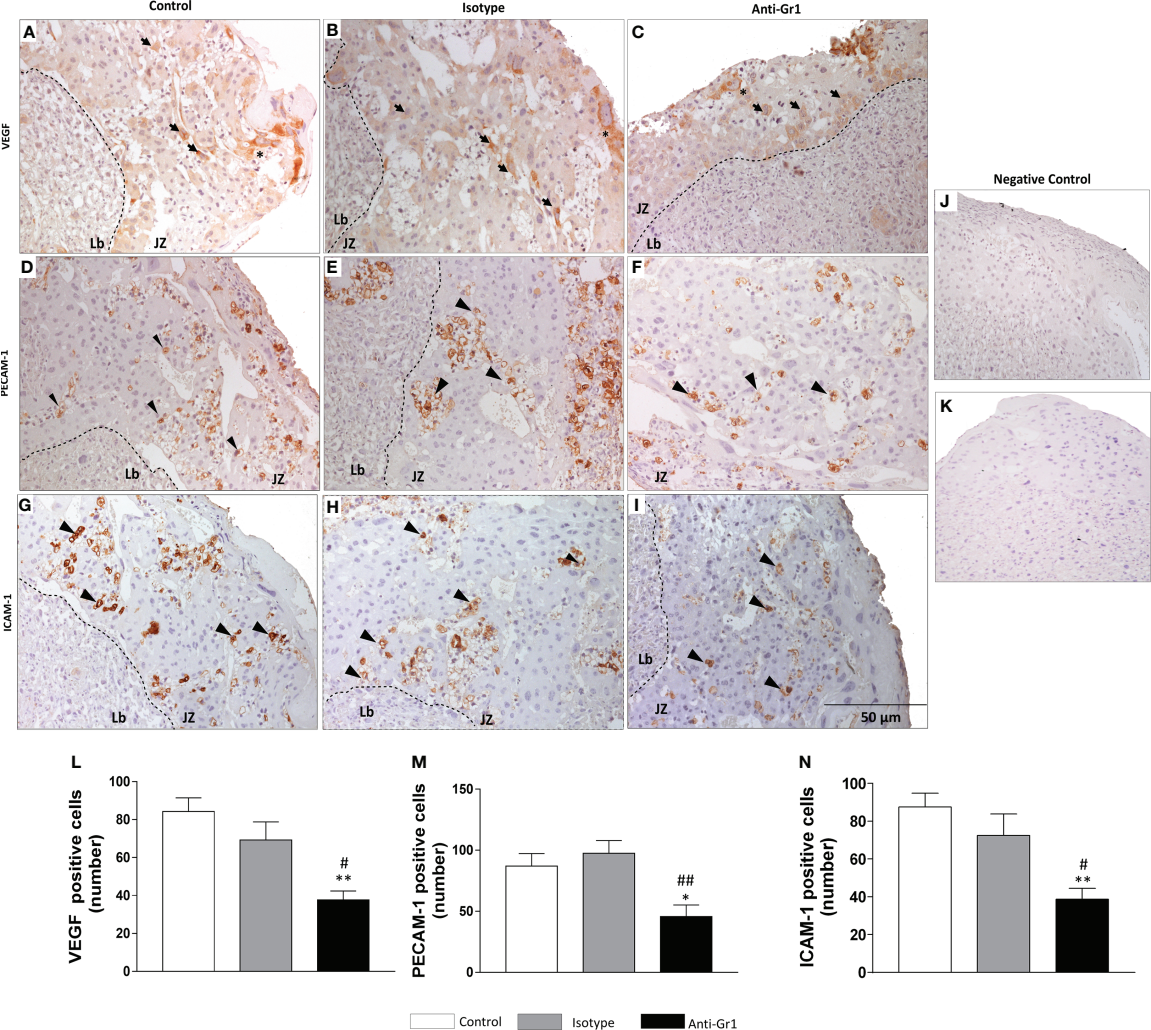
Neutrophil depletion altered angiogenesis and adhesiveness markers in the placenta. Expression and distribution of VEGF **(A-C)**, PECAM-1 **(D-F)**, and ICAM-1 **(G-I)** in the junctional zone of placenta were evaluated by immunohistochemistry. Negative control sections labeled with anti-rabbit **(J)** and anti-goat **(K)** HRP-conjugated antibodies. Count of VEGF **(L)**, PECAM-1 **(M)** and ICAM-1 **(N)** positive cells. *, **p < 0.05; 0.01 in comparison to control group; #, ##p <0.05, 0.01 in comparison to isotype. Data were statistically analyzed using One-Way ANOVA followed by Tukey’s post-test (n= 4 – 6 placentas). Results were expressed as mean ± SEM. Immunoreactive cells for adhesiveness markers are indicated as arrowheads. Asterisks - giant trophoblast cells; TGCs. Arrows - spongiotrophoblast – SpTs. Counterstaining with hematoxylin; embedding paraffin; sections thickness 4 µm. Bars 50 µm.

Since VEGF, PECAM-1, and ICAM-1 are important for angiogenesis and placentation, their expression was assessed in the junctional zone by immunohistochemistry. We verified that VEGF protein was expressed by TGCs and SpTs (**Figures 3A–C**). However, the neutrophil depletion reduced the number of VEGF-positive cells when compared to control/isotype groups (**Figure 3L**). The expression of PECAM- 1 (**Figures 3D–F**) and ICAM-1 (**Figures 3G–I**) was located especially in cells next to maternal vessels, being this pattern was observed for all studied groups (**Figures 3J**, **K** show the negative control). Nonetheless, the number of positive cells for the adhesion markers decreased in the neutrophil-depleted compared to the control/isotype groups (**Figures 3M, N**).

### Neutrophils depletion modified the circulating leukocytes profile and promoted morphologic alterations in the offspring

**Figure 4:**
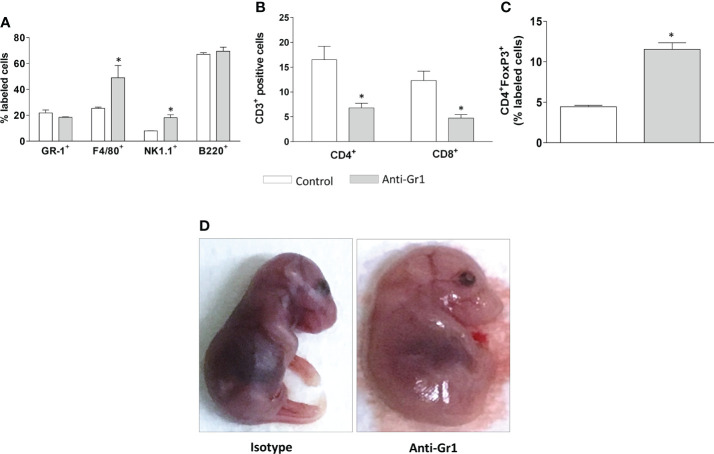
Neutrophil depletion altered the peripheral leukocyte profile and offspring development. On gestational day 18.5, peripheral leukocytes from mice were collected for immunophenotyping of granulocytes (Gr-1), monocytes (F4/80), *natural killers* cells (NK1.1), and mature B cells (B220) **(A)**. T lymphocyte subtypes were divided into CD3+ CD4/CD8 positive cells **(B)** and T regulatory cells, characterized as CD3+CD4+FoxP3+ positive cells **(C)**. The fetuses were carefully removed for gross analyses **(D)**. *p < 0.05 in comparison to the control group. Data were statistically analyzed using One-Way ANOVA followed by Tukey’s post-test (n= 3 – 4 animals/group). Results were expressed as mean ± SEM.

The analysis of circulating leukocytes performed at the end of pregnancy showed the frequency of monocytes (F4/80+) and *natural killers’* cells (NK.1+), which were higher in neutrophil-depleted mice in comparison to control/isotype groups. The frequency of neutrophils (Ly6G+) and B lymphocytes (B220+) was similar for both groups (**Figure 4A**). Furthermore, the circulating CD4+ and CD8+ cells were reduced in the neutrophil-depleted group (**Figure 4B**). However, in the CD4+ population, the frequency of T regulatory cells (FoxP3+) was increased in the neutrophil-depleted group (**Figure 4C**).

Considering that neutrophil depletion affected the placenta structure, we performed a gross evaluation of the offspring. Indeed, neutrophil depletion promoted significant abnormalities, as offspring presented an overall swollen appearance, indicative of generalized edema, and the limbs remained attached to the body (**Figure 4D**). It was also observed that the alterations displayed a pattern among the fetuses (**Supplementary Figure 2A**). The fetal weight was not changed (**Supplementary Figure 2B**). Altogether the gross analyses may indicate that neutrophil depletion evokes delayed fetal development.

## Discussion

Pre-implantation embryos are highly sensitive to environmental conditions during the early development process (27), which includes embryonic epigenome remodeling, and the first cell differentiation. In this phase, blastocyst differentiates into inner cell mass which gives rise to the embryo, and trophectoderm will form the placenta (28).

It is well known that the implantation site resembles an inflammatory response, with a profusion of recruited immune cells (29) into the endometrial stroma and lumen from the blood (30–32). Innate immune cells are the significant population of leukocytes in the uterus at the time of blastocyst implantation. Uterine NK (uNK) cells are the most abundant population, although DCs, macrophages, neutrophils, and mast cells are also present (33). Even though the role of immune cells in pregnancy has been extensively investigated, many questions remain open. Thus, approaches such as immune cell depletion have been applied to better understand the role of the immune system at different phases of pregnancy. In this context, it has been shown the depletion of T regulatory cells in the early phase of pregnancy causes increased fetal resorption, maternal systemic inflammation, enhanced uterine artery resistance to flow and increased nitric oxide modulation of blood pressure (34). Furthermore, macrophages depletion evokes blastocyst implantation failure due to disruption of the luteal microvascular network associated with the decreased production of progesterone, which is essential for establishing pregnancy (35). Recently, the neutrophil’s role in the placenta has been also discussed, as they address their role in the earliest phases of gestation (19, 22). Indeed, we here observed the depletion of neutrophils sustained at the beginning of WOI promoted a reduction of implantation points and compromised the placenta architecture and fetal development, reinforcing the signaling induced by neutrophils is necessary to the implantation of the blastocyst.

Implantation depends on a genetically healthy blastocyst and uterine receptivity (36). However, the immune cells’ repertoire decides whether the uterine endometrium will be receptive or refractive. An immune-competent and receptive endometrium will tolerate and support the trophoblast invasion to initiate the placentation (33). Mouse placenta is organized in the maternal decidua, junctional zone, and labyrinth. The trophoblast giant cells (TGC) and spongiotrophoblast (SpT) are found in the junctional zone and the syncytiotrophoblast in the labyrinth (37). The TGCs help the embryo invade the uterine epithelium and implant it into the endometrium (38). These cells also acquire an invasive phenotype, penetrating deeply into the endometrial stroma and contacting maternal arteries, promoting vascular remodeling, the crucial step that supports the anatomical foundations for placenta development (39). While SpT probably has a structural function and produces several layer-specific secreted factors that may prevent the growth of maternal blood vessels into the fetal placenta (40). Here, morphometric analyses showed the lack of maternal peripheral neutrophils decreased the placenta area due to junctional zone reduction, which presented a decreased TGC and SpT cells number. The reduced junctional zone observed in different experimental conditions has been associated with changing the endocrine environment in the placenta locally and with systemic effects on both fetus and mother (41–43). Also, the control of invasion of trophoblast cells depends on their interaction with immune cells (44, 45). Herein, the neutrophils depletion at the beginning of pregnancy seems to alter the invasion of trophoblast, and consequently impairs the proper placenta development.

Besides morphometric analyses, angiogenic and adhesiveness markers were assessed in the junctional zone. The VEGF-induced signaling controls the angiogenesis-vascularization during placentation. It is expressed in arteriolar smooth muscle, endothelium, trophoblast, uNKs, and decidual cells (46–49). The VEGF expression in the placenta microenvironment has been associated with the modulation of the TGC development and differentiation, and consequent maintenance of the homeostasis of the maternal vascular space in the mouse placenta (50). Additionally, placental VEGF has related to the modulation of metalloproteinase 2 and 9, which play a role in the endometrial tissue remodeling at implantation (51), decidualization (52), and trophoblast invasiveness (53, 54). The imbalance in the VEGF expression is associated with the abnormal placenta and its pathologies such as pre-eclampsia (55). Furthermore, it has been demonstrated that neutrophils at decidua basalis adopt a unique phenotype, expressing high levels of activation markers and angiogenesis-related proteins such as VEGF (13). Although immunohistochemical staining is not the most accurate for quantitative determination, here, we found a reduced number of VEGF-positive trophoblast cells associated with neutrophil depletion during the pre-receptive phase of embryo implantation.

Homotypic and heterotypic cell adhesions are pivotal to blastocyst implantation, and deficiencies in cell-cell interactions impair this process. Immunoglobulins cell adhesions, such as ICAM-1 and PECAM-1, display pivotal roles in immune response and tissue structure, as they are adhesion membrane proteins that cross-link with actin filaments. Also, they are relevant players in intracellular signaling during cell adhesions (56, 57). In pregnancy, ICAM-1 expression on the apical membrane of uterine epithelial cells and on the inner cells of trophoblast formation is enhanced at the time of implantation, which seems to be induced by progesterone secretion (58, 59). Furthermore, ICAM-1 is involved in maternal tolerance maintenance by controlling the secretion of Th2 cytokines (60). By being expressed on endothelial cells, PECAM-1 is highly involved in the angiogenesis process and its up-regulation on the placenta is expected, as novel vessels are required to irrigate the growing fetus. Several pieces of evidence support the role of PECAM-1 on pregnancy failures, as in preeclampsia, cytotrophoblasts fail to express PECAM-1 (54, 61). Mothers down expressing PECAM-1 suffer recurrent implantation failures, in a mechanism depending on the secretion of transforming growth factor β1 induced by PECAM-1 (62), and the impairment of PECAM-1 expression on blastocyst has been pointed out as an emerging marker to successfully assisted transfer embryo (63). Furthermore, PECAM-1 is detected early in the pluripotent inner cell mass of mouse blastocyst, which contains no vascular cells (64) (61), suggesting that it may exert additional roles on blastocyst implantation beyond angiogenesis. Despite the mechanisms displayed by neutrophils needing to be further evaluated, it is possible to infer that the impairment of VEGF, ICAM-1, and PECAM-1 positive cells associated with neutrophil depletion at the earlier phase of pregnancy contributes to placenta and fetus deficiencies shown here.

Many factors are involved in fetal malformation that includes specific genetic defects or congenital abnormalities in the embryo itself, intrauterine infections, and defects in placentation (39, 65). The association between placental dysfunction and fetus alterations has been explored in different contexts. It was shown that the majority of animal lethality found during genetic manipulation was associated with placental dysmorphologies, which were correlated with abnormalities, such as forebrain development, heart chamber, and septum morphology, subcutaneous edema, and overall artery or vein topology (66). Furthermore, alterations in the placenta during preeclampsia cause defective trophoblast invasion, spiral arterial impairment, and maternal decidua remodeling, leading to maternal and fetal complications or diseases (67, 68). Moreover, impairment in the development of the junctional, zone promotes endocrine changes in the placenta environment leading to reduced fetal growth (41, 43). Hence, the placental alterations promoted by neutrophil depletion during the pre-receptive phase of embryo implantation may be associated with the morphological alterations found in the offspring.

Altogether, the data obtained here highlight the relevance of maternal neutrophils in the earliest phase of pregnancy and to sustain placentation and fetus development. The reduced number of neutrophils in the pre-receptive phase led to fetal morphological alterations and an imbalance in the maternal peripheral leukocyte profile. The present data shed light on the intrinsic communication between neutrophils and pregnancy successful; further investigations will provide data to elucidate the fine-tuning mechanisms and signaling triggered by neutrophils during blastocyst implantation.

## Data availability statement

The raw data supporting the conclusions of this article will be made available by the authors, without undue reservation.

## Ethics statement

The animal study was reviewed and approved by Institutional Animal Care and Use Committee from the Faculty of Pharmaceutical Sciences of the University of Sao Paulo.

## Author contributions

Conceptualization, CH, SF, and SS; Data curation, PS and SS; Funding acquisition, SF; Investigation, CH, PS, CG, and SS; Experiments, CH, AS, PS, and SS; Statistical analysis, CH, and SS; Supervision, SF, and SS; Validation, SF, and SS; Writing—original draft, CH, PS, SF, and SS; Writing—review and editing, CH, PS, SF, and SS. All authors contributed to the article and approved the submitted version.

## Funding

This work was supported by FAPESP (Fundação de Amparo á Pesquisa do Estado de São Paulo), grant number 2014/07328-4; SF is a fellow researcher of CNPq (Conselho Nacional de Pesquisa); CH and SS were post-doctoral fellows of CAPES (Coordenação de Aperfeiçoamento de Pessoal de Níel Superior); PS is a PhD fellow of the FAPESP, grant number 2020/14368-3. AS is a Msc fellow of the CAPES.

## Acknowledgments

The authors thank Franciani Rodrigues da Rocha from Núcleo de Pesquisa em Ciências Médicas, Fundação Universidade para o Desenvolvimento do Alto Vale do Itajaí – UNIDAVI; Rio do Sul, SC, Brazil for the statistical analysis support.

## Conflict of interest

The authors declare that the research was conducted in the absence of any commercial or financial relationships that could be construed as a potential conflict of interest.

## Publisher’s note

All claims expressed in this article are solely those of the authors and do not necessarily represent those of their affiliated organizations, or those of the publisher, the editors and the reviewers. Any product that may be evaluated in this article, or claim that may be made by its manufacturer, is not guaranteed or endorsed by the publisher.

## Supplementary material

Supplementary Figure 1 Effects of neutrophil depletion on maternal weight gain, fetuses, and placental weight and index. Pregnant mice were weight on gestational day 1 and before euthanasia on day 18.5 **(A)**. After the cesarean, placenta **(B)** and pregnant utero **(C)** were weighted. The placenta index was based on the ratio of fetus weight in relation to its respective placenta **(D)**. Data were statistically analyzed using test “t” (n = 3 - 6). No significant difference was observed.

Supplementary Figure 2 Effects of neutrophil depletion on the offspring development. Representative image of offspring from control/isotype and anti-Gr1 groups **(A)**. Fetus weight **(B)**.

Supplementary Table 1 Pregnancy index (PI) was calculated considering the of number of females delivering live pups/number of females with evidence of pregnancy*100. Data was statistically evaluated using Fischer Exact Test and results obtained showed there was no statistical association between Isotype/PBS and anti-Gr1 (0.13) as p < 0.05 was considered statistically significant.
