# Supplementary figures and images for: Neutrophil depletion in the pre-implantation phase impairs pregnancy index, placenta and fetus development
Source: Front Immunol. 2022 Sep 29;13:969336. doi: 10.3389/fimmu.2022.969336 (PMC9558710; doi:10.3389/fimmu.2022.969336)

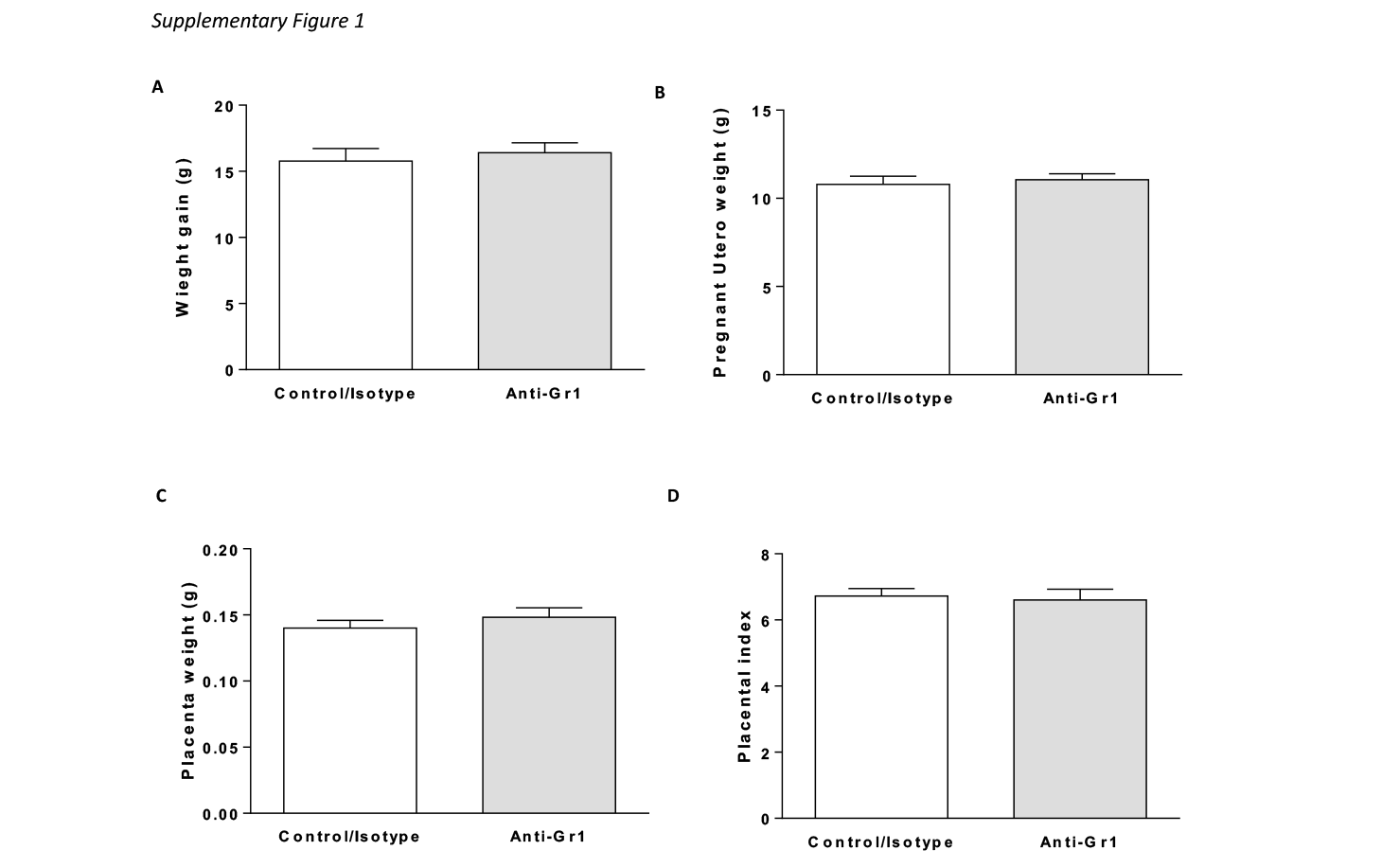

Supplement: Supplementary Figure 1 — Effects of neutrophil depletion on maternal weight gain, fetuses, and placental weight and index. Pregnant mice were weight on gestational day 1 and before euthanasia on day 18.5 (A). After the cesarean, placenta (B) and pregnant utero (C) were weighted. The placenta index was based on the ratio of fetus weight in relation to its respective placenta (D). Data were statistically analyzed using test “t” (n = 3 - 6). No significant difference was observed. [file Image_1.tif]

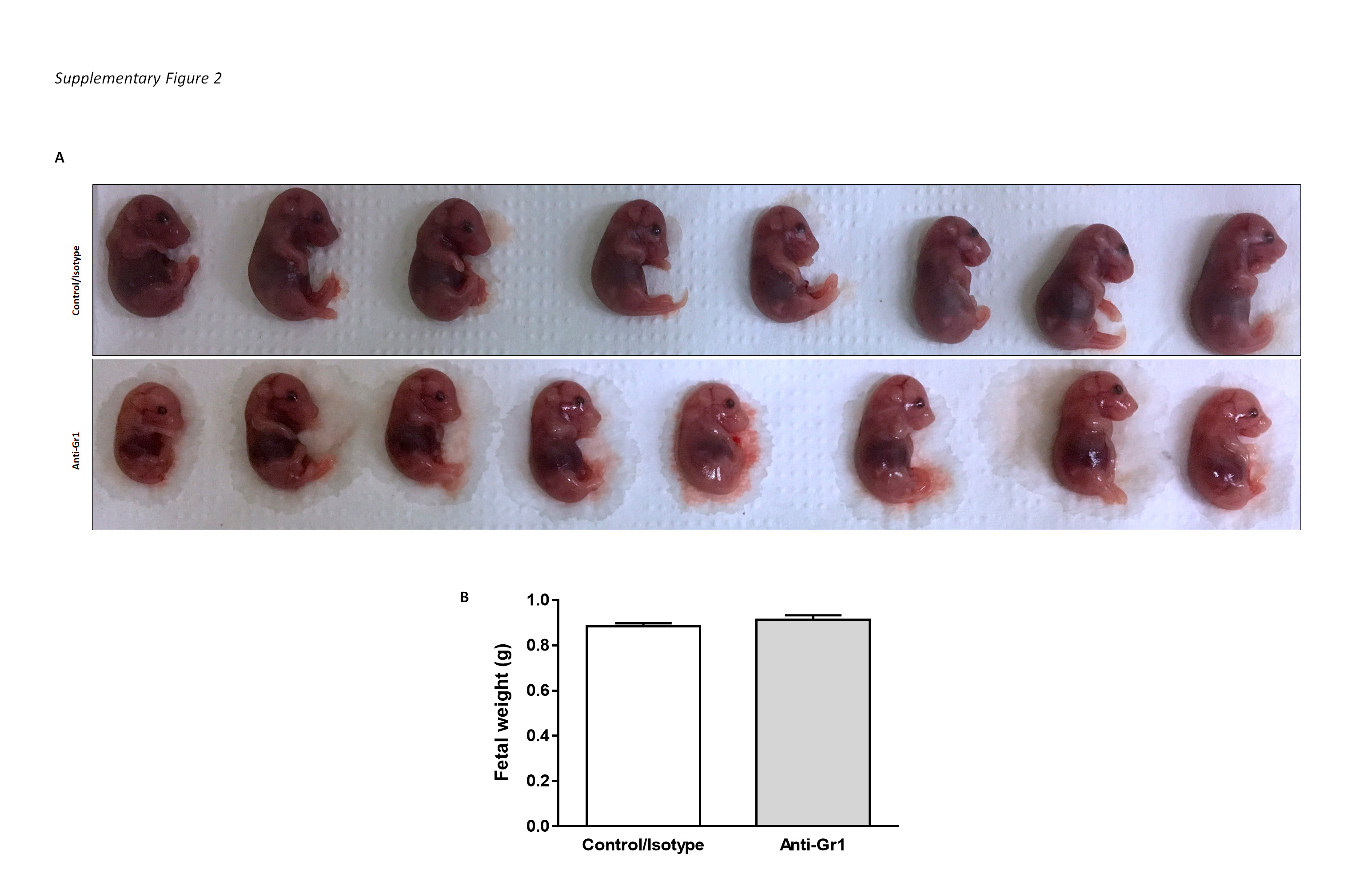

Supplement: Supplementary Figure 2 — Effects of neutrophil depletion on the offspring development. Representative image of offspring from control/isotype and anti-Gr1 groups (A). Fetus weight (B). [file Image_2.jpeg]
