# Supplementary material for: Neutrophil depletion in the pre-implantation phase impairs pregnancy index, placenta and fetus development
Source: Front Immunol. 2022 Sep 29;13:969336. doi: 10.3389/fimmu.2022.969336 (PMC9558710; doi:10.3389/fimmu.2022.969336)
Supplement: Supplementary Table 1 — Pregnancy index (PI) was calculated considering the of number of females delivering live pups/number of females with evidence of pregnancy*100. Data was statistically evaluated using Fischer Exact Test and results obtained showed there was no statistical association between Isotype/PBS and anti-Gr1 (0.13) as p < 0.05 was considered statistically significant. [file Table_1.docx]

|  | **Group** | |  |
| --- | --- | --- | --- |
| **Pregnancy**  **Evolution** | **Isotype/PBS**  **n (%)** | **Anti-Gr1**  **n (%)** | ***p-value*** |
| Pregnancy Failure | 1 (14.3) | 6 (60.0) | 0.13 |
| Pregnancy Successes | 6 (85.7) | 4 (40.0) |  |

**Table 1.** Association between treatments and pregnancy evolution. n: sample size; % percentage. p-value was calculated using Fischer Exact Test and p < 0.05 was considered statistically significant.
